# Cortical tibial osteoperiosteal flap technique to achieve bony bridge in transtibial amputation: experience in nine adult patients

**DOI:** 10.1007/s11751-013-0152-0

**Published:** 2013-01-31

**Authors:** Mauricio Leal Mongon, Felipe Alberto Piva, Sylvio Mistro Neto, Jose Andre Carvalho, William Dias Belangero, Bruno Livani

**Affiliations:** 1 Orthopedics and Traumatology Department, Hospital Estadual de Sumaré, UNICAMP, Rua Jose Augusto Silva 761 apto 31B, Campinas, Sao Paulo zip 13087-570 Brazil; 2 Orthopedics and Traumatology Department, State University of Campinas (UNICAMP), Campinas, Sao Paulo Brazil; 3 Physiotherapy Department, Instituto de Prótese e Órtese (IPO), Campinas, Sao Paulo Brazil

**Keywords:** Amputation, Surgical technique, Tibia, Fibula, Flap

## Abstract

Amputation, especially of the lower limbs, is a surgical procedure that gives excellent results when conducted under the appropriate conditions. In 1949 Ertl developed a technique for transtibial osteomyoplastic amputation which restored the intraosseous pressure through canal obliteration and expanded the area of terminal support through a bony bridge between the fibula and distal tibia. The aim of this study was to investigate the effectiveness of a modification of the original Ertl’s technique in which a cortical osteoperiosteal flap created from the tibia is used to form a bony bridge during transtibial amputation in adults. Nine patients underwent leg amputations with the cortical tibial osteoperiosteal flap technique for reconstruction of the stump. The average duration of follow-up was 30.8 (range, 18–41) months. The post-surgery examination included a clinical examination and radiography. A 6-min walk test (Enright in Respir Care 48(8):783–785, 2003) was performed in the 32nd week after amputation. At 24th week post-surgery, all patients had stumps that were painless and able to bear full weight through the end. The creation of a cortical osteoperiosteal flap from the tibia to the fibula during transtibial amputation is a safe and effective technique that provides a strong and painless terminal weight-bearing stump. This constitutes a useful option for young patients, athletes, and patients with high physical demands.

## Background

Amputation, especially of the lower limbs, can give excellent results when used for correct indications [1]. Despite modern reconstruction techniques and replantation, the preservation of a severely traumatised lower limb or a limb that is affected by painful chronic osteomyelitis usually yields poorer functional results than amputation and prosthetic use [2]. Transtibial amputations result in excellent functional outcomes [3]. In 1949 Ertl developed a technique for transtibial osteomyoplastic amputation which restored the intraosseous pressure through canal obliteration and expanded the area of terminal support by creating a bony bridge between the fibula and distal tibia [4]. The original technique involved the preparation of a periosteum cylinder that was extracted from the tibia with attached bone fragments, which promoted tibiofibular synostosis at the distal extremity of the amputation stump. A major problem with this technique was that it is not always possible to achieve bony bridge formation [5]. Subsequently, variations of the bony bridge have been described [6, 7].

The aim of the present study was to describe a cortical tibial osteoperiosteal flap technique that generates a bony bridge during transtibial amputation of adults. In essence, this is a modification of the original technique described by Ertl.

## Patients and method

### Patients

Between December 2008 and November 2010, nine patients underwent leg amputations with the cortical tibial osteoperiosteal flap technique for reconstruction of the stump. The pre-operative characteristics of the patients are listed in Table 1. The study sample was adult patients who had traumatic and osteomyelitic reasons for amputation. Those under the age of 18 years or with an insufficient tibia length to allow creation of the osteoperiosteal flap (i.e. the same contraindication as that for the original Ertl’s technique) were excluded [8]. None of the selected patients had a systemic comorbidity (e.g. hypertension, diabetes, chronic vascular insufficiency), but three patients were smokers.

**Table 1:** Pre-operative patients data

Patient number	Age at amputation (years)	Gender	Aetiology	Side	Smoker
1	19	Male	Trauma Gustilo IIIC	Right	No
2	46	Male	Chronic Osteomyelitis	Left	Yes
3	27	Male	Trauma Gustilo IIIC	Right	No
4	29	Male	Trauma Gustilo IIIC	Right	No
5	35	Male	Trauma Gustilo IIIC	Right	Yes
6	19	Female	Trauma Gustilo IIIC	Right	No
7	31	Male	Trauma Gustilo IIIC	Right	No
8	18	Male	Trauma Gustilo IIIC	Left	No
9	51	Male	Chronic Osteomyelitis	Left	Yes

There were eight men and one woman (mean age, 30.5 years; range, 18–51 years). Six patients underwent amputation on the right side and three on the left side. All the amputations were unilateral. Seven patients presented with Gustillo & Anderson IIIC open fractures [9, 10], and two patients had chronic painful osteomyelitis of the ankle and hind foot.

The average duration of follow-up was 30.8 (range, 18–41) months. The post-surgery examination included a clinical examination and radiography. A 6-min walk test [11] was performed in the 32nd week after amputation.

This study was carried out with the approval of an Ethics Committee. All patients provided informed written consent in accordance with the World Medical Association Declaration of Helsinki.

### Operative technique

A transtibial amputation using the cortical tibial osteoperiosteal flap technique to create a bony bridge must be performed as distally as possible; the desired level is the osseous equivalent of the muscle–tendon transition of the gastrocnemius muscle (as in the original Ertl’s technique).

All patients received antibiotic prophylaxis (cephalosporin 2 g, administered intravenously within 30 min of the start of the procedure). The surgical procedure begins with two 8-cm longitudinal incisions, one anterolateral and the other posteromedial, starting 2.5 cm above the level proposed for the tibial osteotomy. Distally, these incisions are connected by a circular incision. The two flaps so formed are elevated, keeping the deep fascia and muscular aponeurosis intact. A vertical incision is then made in the deep fascia, just lateral to the tibial crest. At this point, extreme care must be taken to avoid damage to the periosteum. Another vertical incision is made through the deep fascia, in alignment with the fibula. All of the anterior and lateral compartment muscles are removed. Up to this point, both the bones and the interosseous membrane are intact.

**Fig. 1:**
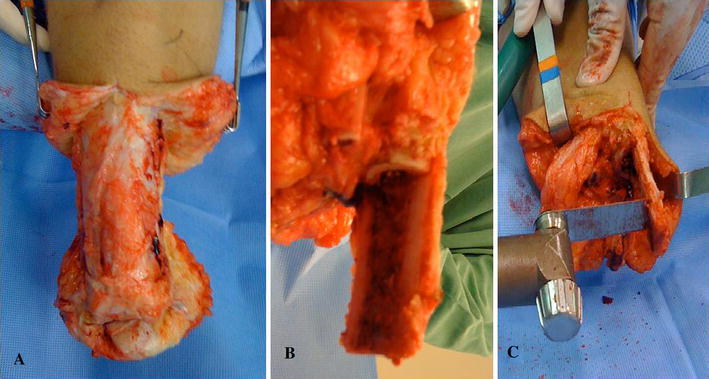
**a** The amputated distal extremity is removed; **b**, **c** an longitudinal ostectomy of the fibular half of the tibia is made

The fibula is sectioned at the final level planned for the tibia while preserving the interosseous membrane. The tibia is sectioned about 8 cm below the fibula, and the amputated distal extremity is removed (Fig. 1a).

The main neurovascular bundle is isolated, and the artery and veins clamped separately; the nerves are sectioned after mild traction to ensure that they will retract proximally as in the original technique of Ertl.

**Fig. 2:**
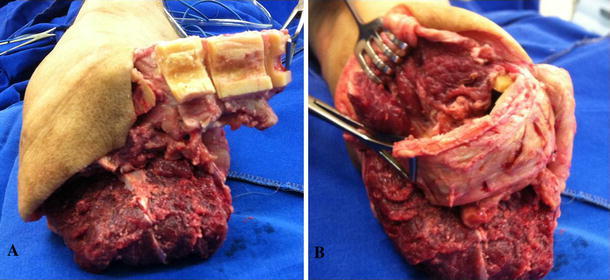
**a** Tibial segmental ostectomy is performed; **b** osteoperiosteal flap is flipped covering both bones

**Fig. 3:**
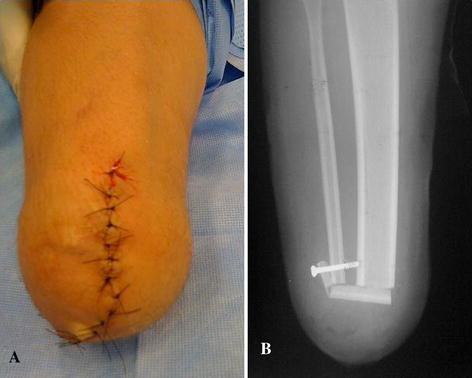
**a** X-ray showing a 3.5-mm screw fixing the distal pedicled strut graft osteoperiosteal flap to the distal fibula; **b** final stump

At the level of the definitive tibial division, a longitudinal osteotomy of the fibular half of the tibia is made and the fibula half removed; the medial half of the tibia remains intact with attached periosteum (Fig. 1b, c). The medial half of the distal tibia is divided into segments while preserving the periosteal layer such that a periosteum-supported strut graft made of these segments is created (Fig. 2a) to allow the osteoperiosteal flap to be flipped to cover both bones. This will eventually connect the tibia to the fibula (future bridge) distally (Fig. 2b). A 3.5-mm screw fixes the distal part of the pedicled osteoperiosteal flap to the distal fibula (Fig. 3a).

When the osteoperiosteal flap is complete, a cushion must be prepared from the two muscle flaps, the aponeurosis, and the remaining intact deep fascia. These must be sculpted properly so as to allow them to be sutured together under mild tension. As described for the original Ertl’s technique, the deep posterior compartment musculature should be resected at the same level as the tibial osteotomy, leaving only the gastrocnemius muscle as a posterior muscular flap. For the tibia, the prominence formed by its crest must be sculpted with round corners without osseous prominences.

The deep musculature enlarges the stump distal volume while the soleus muscle has intramural veins which may contribute to local haematoma formation. At this point, drains should be inserted in the bone and muscular plane. Both long flaps of skin are then cut and sutured under mild tension (Fig. 3b).

The skin suture line is positioned almost perpendicular to the muscle suture line; this helps prevent the formation of undesirable adherent scars among the planes. The surgical dressing used is the same as that recommended for a conventional amputation, that is, an elastic compression dressing that consists of orthopaedic cotton and an elastic bandage. Early knee motion is encouraged.

## Results

The intraoperative procedure had no complications. There were no wound infections, and the incisions healed without complications. The stitches were removed at 3 or 4 weeks post-operatively.

A below-knee prosthesis was adapted to each patient at 7 (range, 6–8) weeks post-operatively. There was no need for stump revision during the entire follow-up period.

**Fig. 4:**
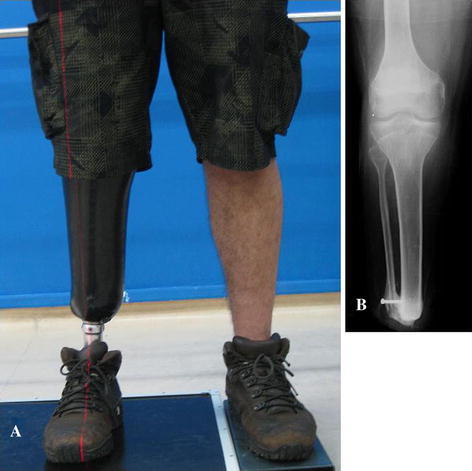
**a** Patient number 3 with prosthesis; **b** final bony bridge

The patients were clinically and radiographically evaluated every 2 weeks. The patients’ stumps were painless and capable of end-bearing weight at an average of 16 weeks post-surgery (range 15–17). Radiographically, all nine patients formed complete bony bridges at an average period of 16 weeks (range, 12–20). The 6-min walk test [11] produced an average result of 312 m (range, 280–340). Each patient was fitted with a prosthesis and subsequently reported a satisfactory quality of life (Fig. 4a, b). Table 2 lists the post-operative information for all the patients.

**Table 2:** Post-operative patients data

Patient number	Follow-up (months)	Bridge formed (weeks)	Time to prosthetisation (weeks)	Terminal weight bearing
1	41	14	7	Yes
2	41	12	7	Yes
3	35	18	8	Yes
4	35	16	7	Yes
5	31	18	8	Yes
6	27	16	6	Yes
7	26	16	7	Yes
8	24	18	6	Yes
9	18	16	8	Yes

## Discussion

In general, the quality of life of a lower-limb amputee with a functional stump is similar to that of an average person [12]. The sooner the patient returns to a daily routine, the greater is the chance of socio-economic re-adaptation [13]. Therefore, a technically well-constructed stump must be the primary objective of the orthopaedic surgeon.

In 1949, Ertl described an amputation technique that employed a bony bridge which, at least, in theory addressed all of the drawbacks of a conventional transtibial amputation [4]. This procedure closes the medullary canal with cortical bone, thereby restoring the intraosseous pressure, blood flow, and vascularisation [14]. The resulting bony bridge increases the terminal area of the stump, makes it more stable, and avoids posteromedial migration of the fibula and consequent funnelling. A larger stump base distributes the weight over a wider area, thereby reducing the pressure [4].

The cortical osteoperiosteal flap, which is a modification of the Ertl’s bony bridge technique, has the advantage of a vascularised flap made of large strut grafts. A larger support area allows for wider distribution of the pressure which reduces the likelihood of pain and increases the weight-bearing ability of the terminal stump. This is particularly important for meeting the greater functional demands of certain patients such as young people, athletes, military personnel, and professionals who exert high levels of physical effort [15].

A drawback of all bony bridge techniques is that the initial level of the lesion cannot lie very proximal to the tibia, as this would prevent the construction of an osteoperiosteal flap of appropriate length. This means that the derivation of a bony bridge is impossible in oncological cases that require a wide surgical margin, as well as in many cases of trauma. In the cases presented here, the amputations were performed on patients who had experienced trauma to the lower limbs requiring early amputation or on patients who required amputation following the development of painful chronic osteomyelitis.

Primary wound closure performed within the zone of injury is a significant predictor of subsequent wound problems, regardless of the amputation technique used [16]. In this series of patients, all the amputations were for trauma and chronic infection and, for safety reasons and to reduce the risk of secondary wound complications (e.g. dehiscence and stump infection), were left open with sterile dressings for 48 h using negative-pressure wound therapy; thereafter, a second examination was performed, at which point the osteoperiosteal flap was constructed and the wound closed [16].

**Fig. 5:**
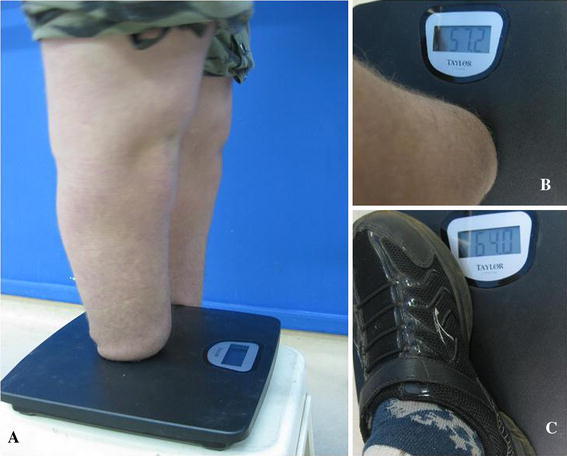
**a** Patient number 1 with terminal full weight bearing; **b** right stump terminal full weight bearing. **c** Total weight of the patient

The intraoperative procedure had no problems or complications. In all patients the wounds healed without complications. A below-knee prosthesis was adapted to all patients, and there was no need for stump revision during the entire follow-up period. All bony bridges were ossified at an average of 16 weeks, similar to the Ertl’s original technique [4]. At the 6-month post-operative follow-up, all patients were able to bear full weight terminally (Fig. 5a–c).

The 6-min walk test is an inexpensive, safe, and easy-to-apply test that can imply level of function with activities of the daily living. It is a way to assess the patient’s functional capacity, monitor effectiveness of a treatment, and establish prognosis, as well as having good correlation with the maximum oxygen consumption [11]. The average result for patients with this technique was 312 m.

## Conclusion

The construction of a cortical tibial osteoperiosteal flap to achieve a bony bridge during transtibial amputation, which represents a modification of the original technique described by Ertl, is a safe and effective procedure that creates a strong, painless, terminal weight-bearing stump. This procedure could be a useful option for young patients, athletes, and patients with high physical demands.
